# Effect of Oxygen on Cardiac Differentiation in Mouse iPS Cells: Role of Hypoxia Inducible Factor-1 and Wnt/Beta-Catenin Signaling

**DOI:** 10.1371/journal.pone.0080280

**Published:** 2013-11-12

**Authors:** Tanya L. Medley, Milena Furtado, Nicholas T. Lam, Rejhan Idrizi, David Williams, Paul J. Verma, Mauro Costa, David M. Kaye

**Affiliations:** 1 Heart Failure Research Group, Baker IDI Heart and Diabetes Institute, Melbourne, Australia; 2 Australian Regenerative Medicine Institute, Monash University, Melbourne, Australia; 3 Centre for Reproduction and Development, Monash Institute for Medical Research, Melbourne, Australia; University of Pécs Medical School, Hungary

## Abstract

**Background:**

Disturbances in oxygen levels have been found to impair cardiac organogenesis. It is known that stem cells and differentiating cells may respond variably to hypoxic conditions, whereby hypoxia may enhance stem cell pluripotency, while differentiation of multiple cell types can be restricted or enhanced under hypoxia. Here we examined whether HIF-1alpha modulated Wnt signaling affected differentiation of iPS cells into beating cardiomyocytes.

**Objective:**

We investigated whether transient and sustained hypoxia affects differentiation of cardiomyocytes derived from murine induced pluripotent stem (iPS) cells, assessed the involvement of HIF-1alpha (hypoxia-inducible factor-1alpha) and the canonical Wnt pathway in this process.

**Methods:**

Embryoid bodies (EBs) derived from iPS cells were differentiated into cardiomyocytes and were exposed either to 24 h normoxia or transient hypoxia followed by a further 13 days of normoxic culture.

**Results:**

At 14 days of differentiation, 59±2% of normoxic EBs were beating, whilst transient hypoxia abolished beating at 14 days and EBs appeared immature. Hypoxia induced a significant increase in Brachyury and islet-1 mRNA expression, together with reduced troponin C expression. Collectively, these data suggest that transient and sustained hypoxia inhibits maturation of differentiating cardiomyocytes. Compared to normoxia, hypoxia increased HIF-1alpha, Wnt target and ligand genes in EBs, as well as accumulation of HIF-1alpha and beta-catenin in nuclear protein extracts, suggesting involvement of the Wnt/beta-catenin pathway.

**Conclusion:**

Hypoxia impairs cardiomyocyte differentiation and activates Wnt signaling in undifferentiated iPS cells. Taken together the study suggests that oxygenation levels play a critical role in cardiomyocyte differentiation and suggest that hypoxia may play a role in early cardiogenesis.

## Introduction

Cardiomyogenesis from specific progenitor cells is critically dependent on the presence of a tightly regulated biochemical and mechanical microenvironment. An appropriate level of tissue oxygen tension is one of the most critical factors for the formation of the heart and its subsequent structural development. In this context, hypoxia is recognized to cause a range of cardiac malformations, including myocardial and valvular hypoplasia [1]–[3]. At the cellular level, hypoxia-inducible factors are one of the pivotal transcriptional regulators of the hypoxic response. Specifically, hypoxia induced genes, including HIF-1, HIF-2 and vascular endothelial growth factor (VEGF), play important roles in the hypoxia-dependent signaling for the development of coronary vessels, myocardial structure and optimal cardiac performance [4].

Hypoxia-inducible factors (HIFs) sense and adapt to low oxygen tension. HIFs are a family of transcription factors consisting of two subunits: the oxygen sensitive alpha subunit, and the constitutively expressed beta subunit, which binds to all HIF alpha combinations (HIF-1alpha, HIF-2alpha, HIF-3alpha, IPAS and NEPAS). Both HIF-1 and HIF-2 protein complexes are expressed in cardiac tissue [5], with a larger body of evidence demonstrating the role of HIF-1alpha expression in myocardial remodeling and coronary vessel formation. Under normoxic conditions HIF-1alpha has a half-life of less than 5 minutes [6], [7], however under hypoxic conditions HIF-1alpha is stabilized and translocated to the nucleus where it dimerizes with HIF-1beta subunits [8] to activate genes that possess hypoxic response elements. Over 70 genes have been shown to respond to hypoxic conditions, including various genes associated with angiogenesis and inhibition of apoptosis and cardiogenesis [9], [10]

In regard to cardiac development specifically, HIF-1alpha knockout mice are embryonic lethal by 11 days post-coitum (dpc) with diverse cardiac anomalies, including cardiac bifida, abnormal looping, abnormal remodeling of the outflow tract, blood vessel deformities and mesenchymal cell death [2], [11]. In the developing cardiomyocyte, HIF-1-dependent activation has been reported to have a dual role by regulating genes associated with vessel formation and myocardium survival, as well as inducing expression of pro-apoptotic genes [12] and hypertrophic markers [13]. As an earlier response to hypoxia, HIF-1alpha has been shown to directly regulate cardiotrophin-1, which exerts a protective function in cardiomyocytes [14]. In contrast, chronic hypoxia has been previously shown to increase apoptotic Caspase-3 in fetal rat hearts [15]. Thus HIF-1alpha plays a role in promoting both survival and cell death of cardiomyocytes depending on the timing and duration of hypoxic conditions. The mechanism underlying the relationship between HIF-1α and cardiogenesis is not well understood. However, based on recent studies showing that HIF can directly interact with the Wnt signaling target beta-catenin [16] and that Wnts may be involved in the response to hypoxia during development in other cell types [17], we hypothesized that the Wnt pathway may be involved in modulating a cardiogenic response to hypoxia during differentiation.

Therefore, in this study we investigate the role of transient hypoxia on the differentiation of embryoid body (EB)-derived cardiomyocytes obtained from mouse induced pluripotent stem (iPS) cells and on the expression of genes in the Wnt pathway. The observations made in the present study may also provide insights into the causation of hypoxia induced cardiac developmental abnormalities, whilst also providing potential information related to the role of Wnt signaling during the generation of mature cardiomyocytes from iPS cells.

## Results

### Generation and characterization of iPS cell lines from skeletal muscle

**Figure 1:**
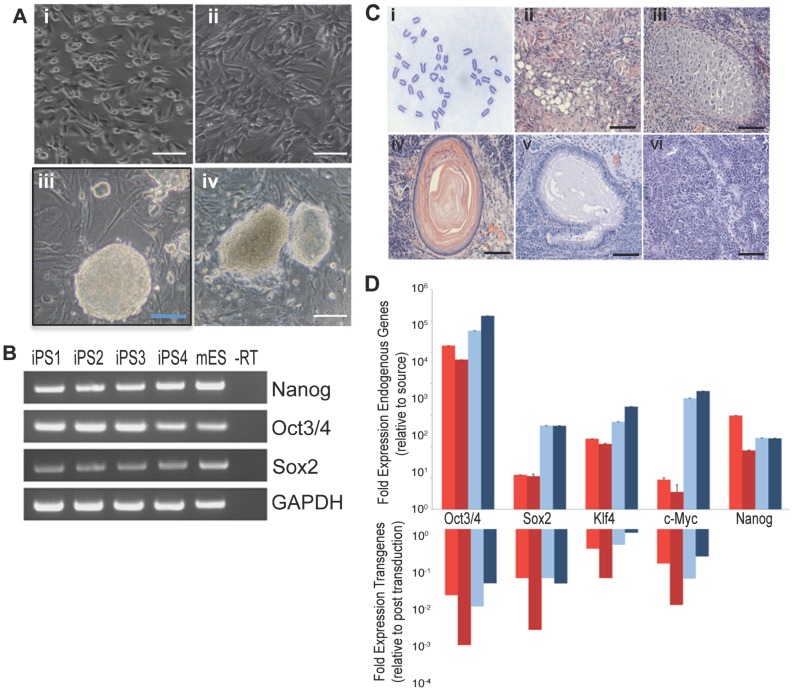
Generation of iPS lines from skeletal muscle. (A). Muscle progenitors (i) and fibroblasts (ii) were isolated from skeletal muscle and used for reprogramming experiments. Four Representative iPS colonies obtained from muscle progenitor (iii, n = 2) and fibroblasts (iv, n = 2). (B). RT-PCR showing endogenous expression of pluripotency genes Nanog, Oct3/4 and Sox2 in reprogrammed iPS colonies. (C). Characterization of chromosomal content shows normal karyotyping (i) and differentiation in tissues from all 3 germ-layers endoderm, mesoderm and ectoderm (ii-adipocyte; iii-cartilage; iv-epidermis; v-ciliated epithelium; vi-neural rossettes) were identified in all 4 lines. (D). Quantification of expression of pluripotency genes after reprogramming for the 4 iPS lines generated, shown in different colors. Scale bar  =  50 um.

For the present studies, iPS cells were derived from mouse skeletal muscle progenitor cells (Figure 1A – panel i) and skeletal muscle fibroblasts (Figure 1A – panel ii) and were generated by retroviral transduction with the classic combination of Yamanaka transcription factors Oct4, Klf5, c-Myc and Sox2 [21]. Following reprogramming, colonies showed morphology comparable to mouse embryonic stem (ES) cells (Figure 1A – panels iii and iv), and four different lines were chosen for further analysis. The molecular signature of reprogrammed colonies was validated by RT-PCR and as expected showed expression of endogenous embryonic stem cell makers Nanog, Oct3/4 and Sox2 in all four lines (Figure 1B). Up-regulation of endogenous pluripotency genes and down-regulation of viral factors initially used for transduction were also assessed by quantitative PCR (Figure 1D). In addition, lines displayed normal karyotyping (Figure 1C – panel i) and were capable of forming tissues derived from all three embryonic germ layers in teratoma assays (Figure 1C – panels ii to vi). These data confirmed successful iPS reprogramming from both skeletal muscle cellular sources.

### Effects of hypoxia on cardiomyocyte differentiation and maturation from iPS cells

**Figure 2:**
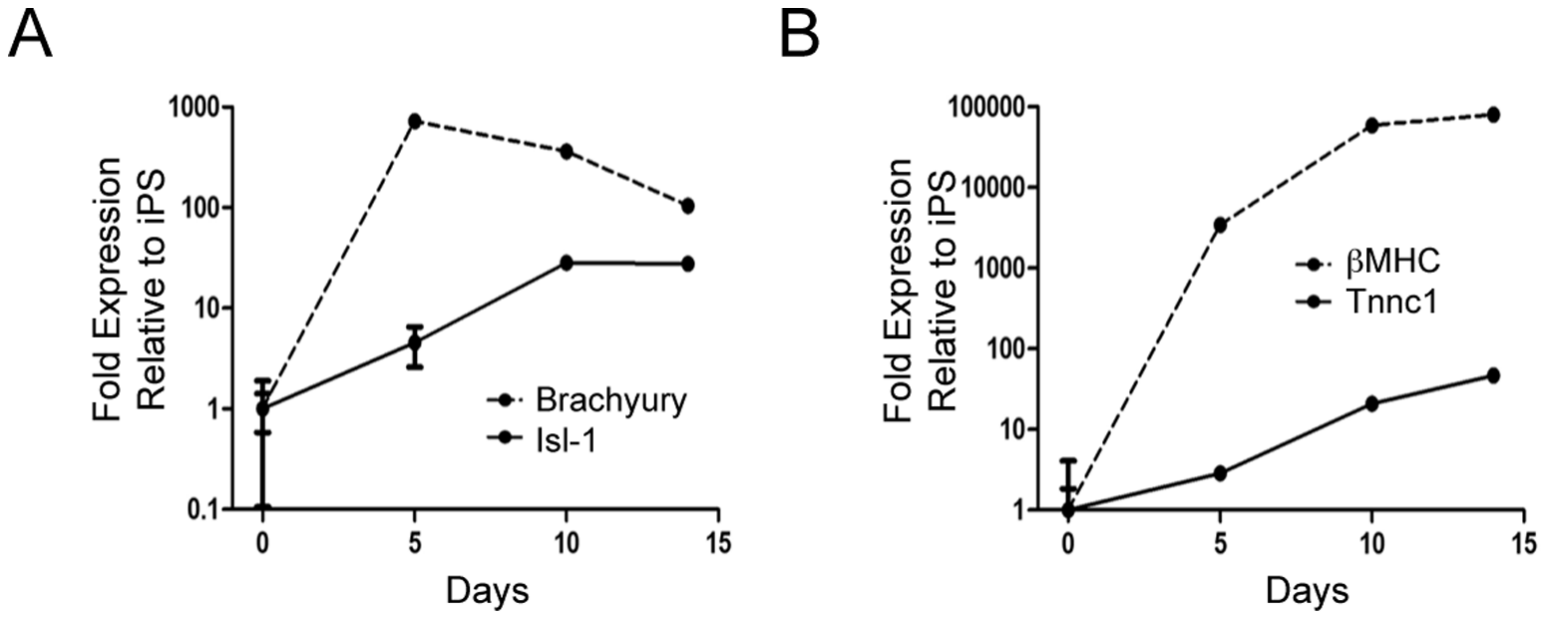
Time-course for expression of mesodermal marker brachyury and the cardiac progenitor marker Isl-1 (A) or for expression of differentiation/structural markers for cardiomyocytes (B) upon EB differentiation under normoxic conditions (n = 4, performed in 2 independent experiments).

iPS cells were differentiated down the cardiac lineage by formation of EBs upon removal of leukemia inhibitory factor (LIF). Following 14 days of culture under normoxic conditions, 59±2% of EBs contained elements exhibiting spontaneous contractions. In parallel with the functional characterization, we evaluated the temporal expression pattern of the cardiac progenitor marker Islet-1 (Isl1), the early mesodermal transcription factor, Brachyury, and the mature cardiomyocyte markers troponin C type 1 (Tnnc1) and beta myosin heavy chain (beta-MHC) over the 14 days of normoxic culture. As shown in Figure 2, expression of Isl-1 initially increased upon EB aggregation, followed by a decline in expression as cells matured and began to beat. As, expected, this later stage coincided with the expression of mature cardiomyocytes markers troponin C type 1 (Tnnc1) and embryonic beta myosin heavy chain (beta-MHC), which steadily increased through differentiation into beating EBs (Figure 2).

**Figure 3:**
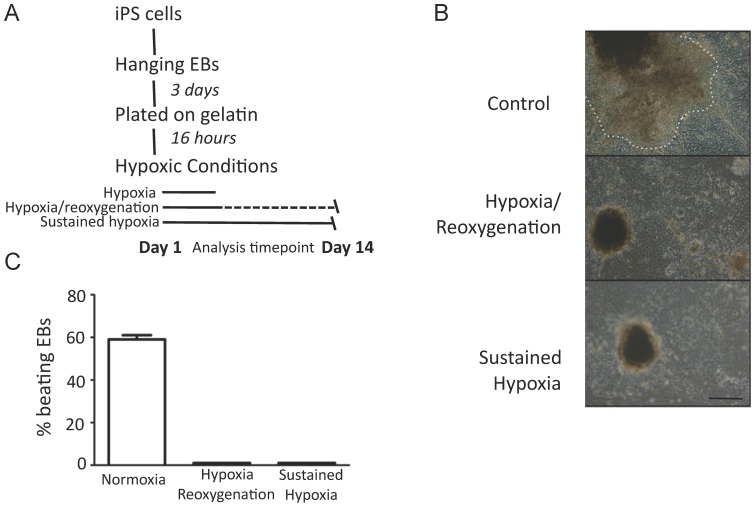
Diagramatic representation of the experimental approach for studies into the effects of hypoxia (A). Representative images of EBs under varying conditions, with 50(B). Observed beating rate (C) (n = 4, performed in 2 independent experiments).

**Figure 4:**
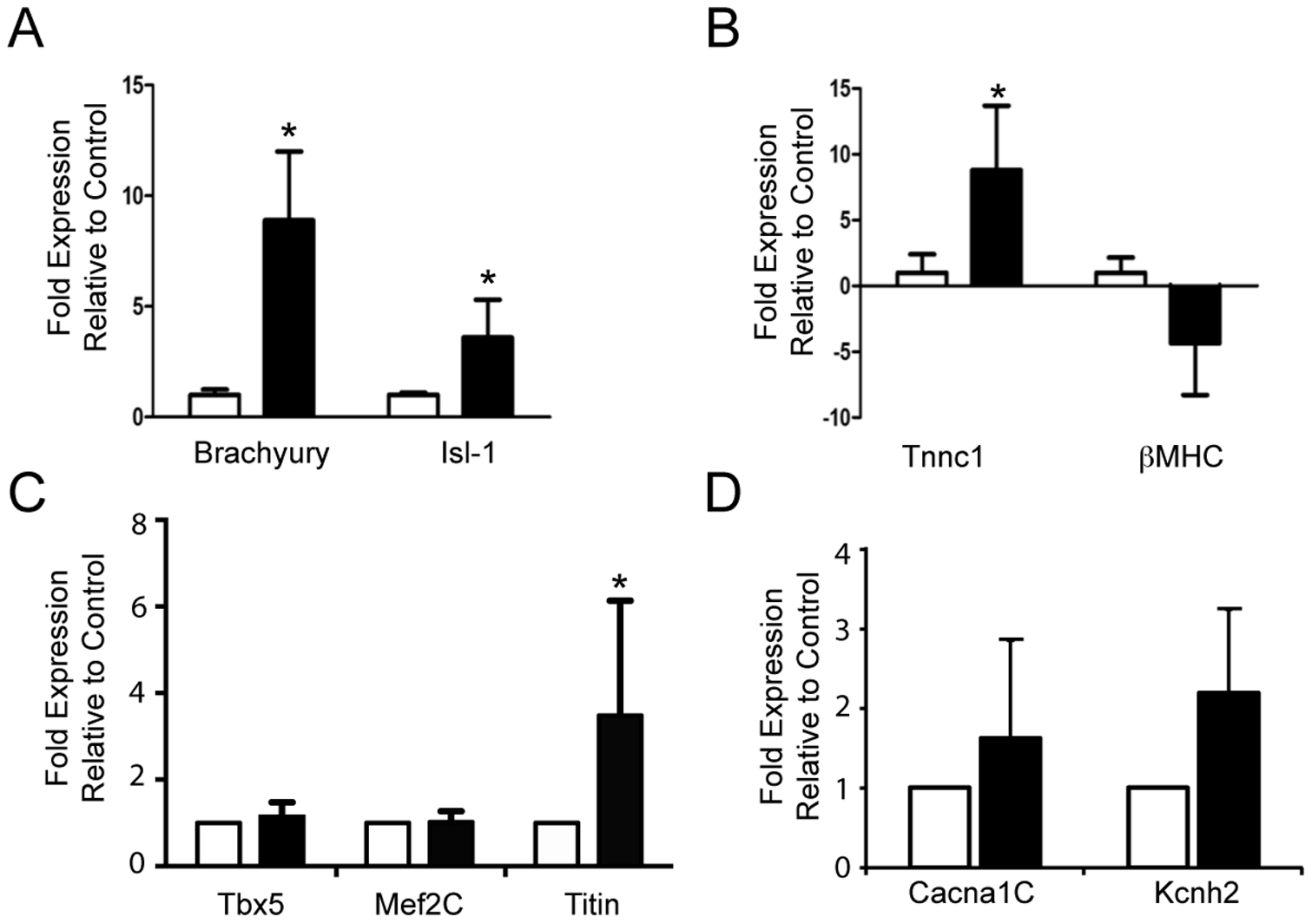
Bar graphs show expression of (A) the mesodermal marker brachury and cardiac progenitor marker Isl-1, (B) differentiation/structural markers for cardiomyocytes, (C) HIF-1alpha target genes Tbx5, Mef2c and titin and (D) ion channels Cacna1c and Kcnh2 upon EB differentiation for 14 days under normoxic (white bars) conditions or following 24 h hypoxia initially followed by normoxia for 13 days (black bars). (* p<0.05; n = 4, performed in 2 independent experiments).

We next aimed to determine whether exposure to short term (24 h) periods of hypoxia influenced the capacity of iPS cells to differentiate into mature cardiomyocytes. In contrast to the development of contractile EBs under normoxic conditions as described above, EBs exposed to an initial 24 hr period of hypoxia followed by 13 days of normoxia failed to develop any evidence of contractile activity at the 14 day timepoint (Figure 3). To investigate the molecular correlate of this observation, we examined the expression of Isl-1, Brachyury, Tnnc-1 and beta-MHC. EBs exposed to transient hypoxia followed by prolonged normoxia demonstrated significantly greater expression of Brachyury (p = 0.01) and Isl-1 (p = 0.04) as shown in Figure 4A. When compared to control, EBs exposed to hypoxia followed by normoxia interestingly showed greater Tnnc1 expression (p = 0.02), whilst beta-MHC expression tended to be lower (p = 0.07, Figure 4B). In conjunction we examined the expression of the HIF-1alpha target genes Mef2c, Tbx5 and titin. As shown in Figure 4C expression of Mef2c and Tbx5 were unchanged at the 14 day timepoint whilst titin expression was increased although with considerable variability. Consistent with the hypoxic induction of a spontaneously beating phenotype, the expression of relavent ion channels was increased as shown in Figure 4D

### Effects of hypoxia on HIF-1α, beta-catenin and Wnt expression

**Figure 5:**
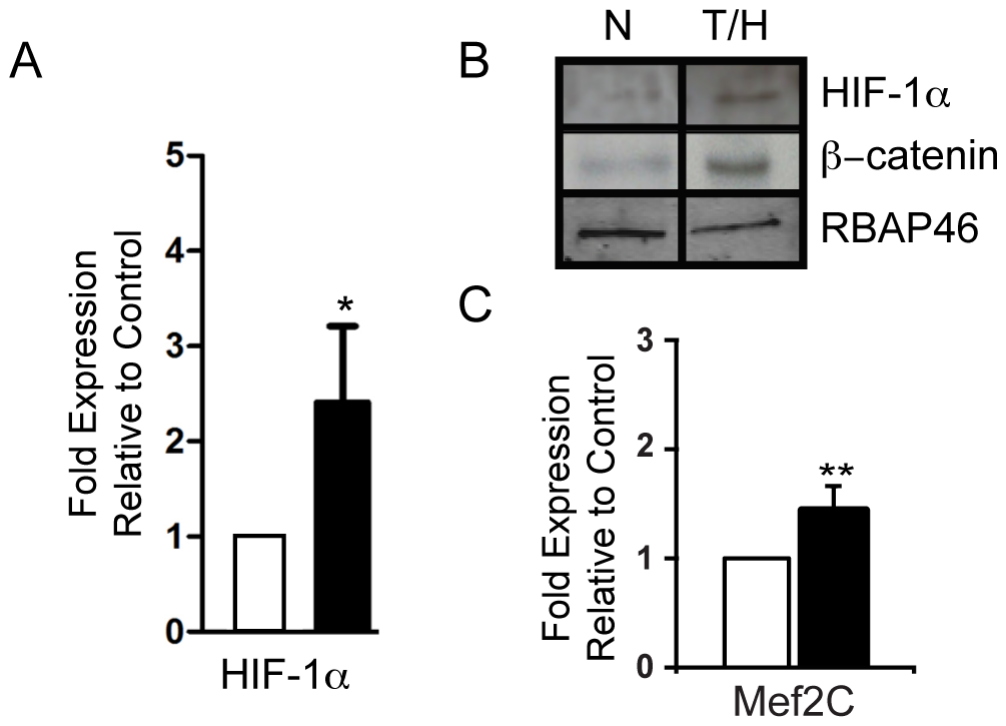
Effects of hypoxia on HIF-1alpha and beta-catenin expression. A. Quantitative PCR showing significant increase of HIF-1α expression following 24 hours hypoxia alone compared to normoxic control. B. Representative Western blots of nuclear extracts from EBs, following 24 h or normoxia or hypoxia. Protein loading was examined by Western blotting against the nuclear protein RBAP46. C. Quantitative PCR showing significant increase of Mef2c expression following 24 hours hypoxia alone compared to normoxic control. All qPCR experiments (n = 4) were performed in 2 independent experiments.

Since hypoxia inducible factor -1 (HIF-1alpha) has been previously shown to be essential for the hypoxic response in heart development [4], [22], we sought to determine the effect of hypoxia on HIF expression in iPS derived EBs. After exposure to hypoxia early in differentiation, EBs showed increased mRNA expression of HIF-1α (Figure 5A, p = 0.03) at 24 hours. We next sought to investigate whether this correlated with the accumulation of nuclear HIF-1alpha and beta-catenin. As shown Figure 5B, hypoxia was associated with accumulation of both HIF-1alpha and beta-catenin in nuclear extracts.

**Figure 6:**
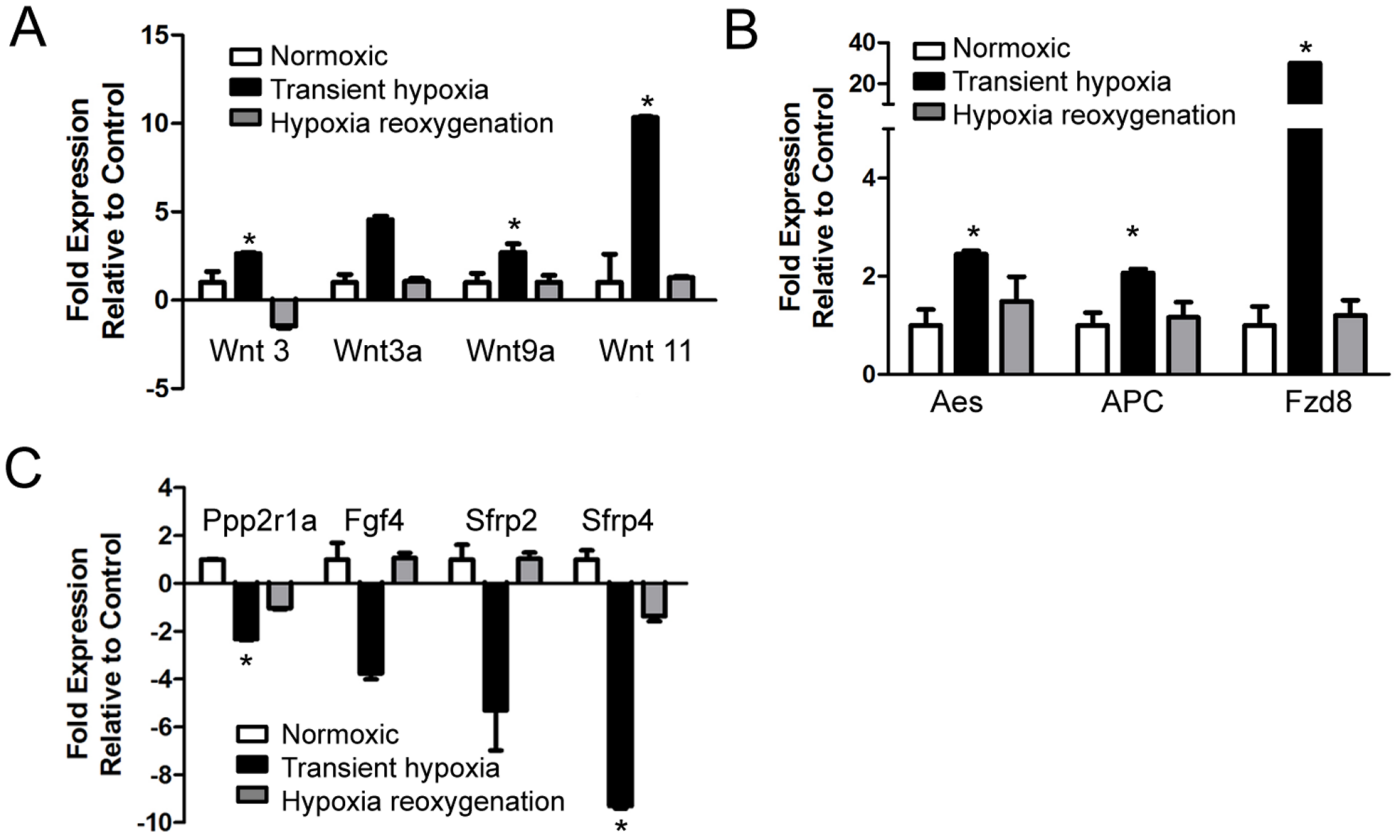
Effects of hypoxia and hypoxia/reoxygenation on Wnt and wnt-related genes in differentiating cardiomyocytes. Bar graphs showing expression of ligands of the canonical Wnt pathway (A), Wnt targets (B) and negative regulators (C). (*p<0.05; n = 4, performed in 2 independent experiments).

Expression of members of the canonical Wnt pathway were significantly upregulated in response to transient hypoxia as shown in Figure 6A. After return to normoxia, most Wnts returned to baseline expression. Furthermore, after exposure to hypoxia there was a marked reduction in the expression of negative regulators/inhibitors of Wnt signaling in EBs compared to those cultured in normoxic conditions, including Protein phosphatase 2, regulatory subunit a (Ppp2r1a, p<0.001) and secreted frizzled-related protein 4 (Sfrp4, p = 0.04) (Figure 6B). The Wnt inhibitors secreted frizzled-related protein 2 (Sfrp2) and fibroblast growth factor 4 (Fgf4) also showed decreased expression, although changes were not statistically significant from controls.

We also examined the temporal pattern of changes in the expression of Wnt signaling targets in response to transient hypoxia and to subsequent normoxia. As shown in Figure 6C, hypoxia caused a significant increase in the mRNA expression of the amino-terminal enhancer of split (Aes, p = 0.01), adenomatosis polyposis coli (Apc, p = 0.01), with a >30 fold increase in frizzled homolog 8 (Fzd 8, p = 0.007). In addition there were significant increases in the gene expression of the beta∼catenin activators Lef-1 (p = 0.001) and Tcf-1 (p = 0.003).

## Discussion

Extensive data indicate that exposure to hypoxia during fetal development has the potential to cause abnormal heart morphology and function [4]. Hypoxia has also been shown to lead to a reduction in fetal cardiomyocyte proliferation together with increased apoptosis [15]. In addition to effects of hypoxia during development, studies by Barker and colleagues suggest that exposure to hypoxia during development may lead to cardiovascular remodeling, ultimately predisposing to aging related cardiovascular diseases [23], [24]. The precise mechanism for the effects of hypoxia on development are unclear, however it is likely that HIFs are involved given previous data linking altered HIF expression to cardiac malformations [1]–[3]. The mechanism underlying the relationship between HIF-1alpha and cardiogenesis is also not well understood, however recent studies have shown that HIF can directly interact with beta-catenin [16] the downstream target of canonical Wnt signaling which is linked to stem cell differentitation.

In the present study, we employed an iPS based approach to investigate the mechanism by which hypoxia influences cardiomyocyte development. In particular we wished to determine whether even short periods of hypoxia might influence cardiomyocyte differentiation from precursors, and to evaluate the potential role of altered Wnt expression. We demonstrated that a short period of hypoxia followed by a return to normoxic conditions lead to a failure of the development of a contractile phenotype in iPS derived EBs. In association this was accompanied a significant prolongation of the expression of the early developmental markers Isl1 and Brachyury, compared to EBs that had undergone differentiation in normoxic conditions.

In contrast with the findings in our study, it has previously been suggested that hypoxia may exert a positive role for on cardiomyocyte differentiation albeit in mouse ES cells (mESCs), in a Cripto-1 dependent manner [25]. As such, our findings have added clinical methodological relevance in regard to the potential derivation of cardiomyocytes from patient and tissue specific iPS cells.

Our study sought to explore the possibility that hypoxia early in cardiomyocyte development may lead to activation of HIF-1α with potential subsequent effects on Wnt gene expression. This hypothesis was based on previous studies showing that distinct stem cell populations exist in hypoxic niches where HIF-1alpha directly regulates the Wnt/beta-catenin pathway to maintain pluripotency [17]. In particular, hypoxic induction of the Wnt pathway mediated by HIF-1α was selective to multipotent neuronal cells, while the same induction was not observed in differentiated neuronal cells [17]. In this context, Wnts are a complex system of growth factors involved in various intracellular signaling pathways that regulate a diverse range of cellular functions in developing cardiomyocytes. They are required for mesodermal induction [26], [27], proliferation and expansion of cardiac progenitor cells. Terminal differentiation of cardiac cells follows a coordinated downregulation of Wnt signaling [28]. Thus, manipulation of Wnt signaling can either positively or negatively affect the derivation of cardiomyocytes dependent on cell context and duration of signaling stimulation or inhibition [29].

We demonstrated a complex pattern of altered Wnt expression following hypoxia, which was accompanied by downregulation of key inhibitors, together favoring a lack of differentiation. Consistent with activation of canonical Wnt signaling and possibly a direct interaction with of HIF [16], we observed a translocation of beta-catenin to the nuclear fraction. Nuclear beta-catenin regulates gene transcription by interacting with Wnt target and activator genes Tcf-1/Lef-1, which we also found to be upregulated in the setting of hypoxia. Interestingly, HIF-1alpha has been shown to prevent the differentiation of hES cells, where it is associated with the up-regulation of pluripotency genes Nanog and Oct-4 [30]. Other studies focused upon the differentiation of mESCs down a vascular lineage have suggested that hypoxic conditions promote differentiation to endothelial cells [31], [32] and similarly hypoxic pre-conditioning of adipose derived stem cells has also been shown to improve their survival and promote angiogenesis after ischemia [33]. In the present study, in association with conversion a more contractile phenotype there was an increase in the expression of titin which is a known target of HIF-1alpha [34]. The expression of other target genes was not apparent, likely suggesting the transient and temporally different nature of gene induction by hypoxia

One of the striking findings of our study was that exposure to relatively short term hypoxia lead to a long term failure of development of a contractile phenotype. The prolonged appearance of immature markers would not necessarily directly explain this finding but are consistent with a failure of progressive differentiation to a contractile phenotype. We did observe a downregulation of the contractile protein beta∼MHC, which may have been a contributing factor, although this was opposite to Tnnc1 expression. We did not specifically explore the reason for the difference between beta∼MHC and Tnnc1 expression, however it is likely that this may reflect different temporal patterns of expression during development and differing expression in cardiomyocytes of varying phenotype. In addition to the modification of contractile protein expression, hypoxia also initiated the expression of key plasma membrane Ca^2+^ and K^+^ channels.

Taken together our study provides a number of important findings relating to the substantial long term alteration in cardiomyocyte development after hypoxia and the potential role of HIF-1alpha and Wnt signaling. Specifically targeted molecular or pharmacologic approaches will be required to further delineate the exact mechanism for this process.

## Methods

### Animals

All animal experimentation was conducted with protocols and ethics approved by the Monash Medical Centre Animal Welfare Committee and the Alfred Medical Research and Education Precinct (AMREP) Animal Experimentation Ethics Committee under the guidelines of the National Health and Medical Research Council of Australia. Every

### Cell culture – Primary cell lines

The gastrocnemius muscle was removed from a C57/Bl6 mouse. Skeletal muscle myoblast progenitors and skeletal fibroblasts cultures were established using a modification of previously established protocols [18], [19]. Briefly, after removal of vessels and connective tissue muscle was dissociated using collagenase/dispase (0.1 U/ml, Sigma). Fibroblasts from the dissociated muscle were adhered to plastic and maintained in Dulbecco’s modified Eagle’s medium (DMEM, Invitrogen) supplemented with 15% fetal calf serum (FCS, Invitrogen) 2 mM L-Glutamine (Invitrogen) and 50 U/ml penicillin, 50 mg/ml streptomycin (Invitrogen). Myotubes were filtered from the dissociated muscle using a100 µm filter (Millipore) and remaining myoblasts were grown on collagen-coated plates in DMEM supplemented with 20% FCS, 4 mM L-Glutamine, 50 U/ml penicillin, 50 mg/ml streptomycin and 0.2 ng/ml basic fibroblast growth factor (bFGF, Invitrogen).

### Derivation of iPS cells

Primary cultures were retrovirally reprogrammed into iPS cells via the retrovial induction of Oct4, Sox2, c-Myc and Klf4 expression as previously described [20]. iPS colonies were cultured on dishes coated with gelatin and irradiated mouse embryonic fibroblast (mEF) feeder layers. iPS cells were maintained in DMEM, 15% ES-FCS (Invitrogen), 2 mM L-Glutamine, 100 uM nonessential amino acids (Invitrogen), 50 uM 2-mercaptoethanol (Sigma-Aldrich), 50 U/ml penicillin, 50 mg/ml streptomycin and 1000 U/ml leukaemia inhibitory factor (LIF, Chemicon). All iPS cells were cultured at 37°C, 5% CO_2_ and passaged using 0.05% trypsin-EDTA (Invitrogen). Phase contrast images of somatic cell lines and iPS colonies were acquired using an Olympus CKX41 microscope.

### Chromosomal analysis

Actively growing iPS cell lines were treated with 10 mM of BrdU (Sigma-Aldrich) for 14 hours followed by Karyomax Colcemid (GIBCO) for a further 4 hours to arrest cells in metaphase. The cells were then collected following trypsinization and lysed in 0.56% KCl before fixation in Methanol:Acetic Acid (3∶1). The cell nuclei were then dropped on glass slides from a height of 0.3 meters and stained with Lieshman Stain (Sigma-Aldrich) for chromosomal count analysis. At least 20 metaphase spreads were counted per cell line.

### Teratoma formation

Briefly, 1–2×10^6^ iPS cells (at passage 3) in a 50 µl volume were injected into hind leg muscle of SCID mice using a 1 ml syringe fitted with a 26–31 needle. After 4 weeks, teratomas were dissected, fixed in 4% paraformaldehyde, embedded in paraffin, sectioned at 5.0 um and stained with haematoxylin and eosin (Sigma). Sections were examined on an Olympus BH2 microscope for cell representative of the three germ layer lineages and images acquired using ImagePro Plus software (Adept Electronic Solutions Pty Ltd, Moorabbin, Australia).

### In vitro differentiation

Embryoid bodies (EBs) were formed by the hanging drop method using differentiation medium containing DMEM, 15% ES-FCS (Invitrogen), 2 mM L-Glutamine, 100 uM non-essential amino acids (Invitrogen), 50 uM 2-mercaptoethanol (Sigma-Aldrich), 50 U/ml penicillin, 50 mg/ml streptomycin. Briefly, iPS cells were dissociated and 10 ul droplets (each containing 800 cells) were hung upside down for 2 days. After an additional 2 days in suspension on ultra-low-binding dishes (Corning) rounded EBs were seeded onto gelatin-coated dishes and media changed every second day for 14 days.

### Hypoxic conditions

Sixteen hours after EBs were seeded onto gelatin, cells were exposed to hypoxia (3% O_2_, balanced with N_2_ for 24 h) in an incubator under hypoxic or control conditions. At 24 h samples were collected as appropriate for RNA or protein extraction. A further series of samples were exposed to hypoxic conditions for 24 h and then returned to normoxia and cultured for a total of 14 days.

### Gene expression analysis: RT-PCR and real-time RT-PCR

For RT-PCR and real-time RT-PCR total RNA was extracted and stored at -70°C. cDNA was synthesized from one microgram of RNA according to the manufacturer’s instructions (Applied Biosystems). PCR arrays were performed on cDNA (SABiosciences). Real-time RT-PCR amplifications were performed with SYBR® Green PCR Master Mix (Applied Biosystems) with samples prepared in triplicate and gene expression normalized to 18S rRNA as an endogenous control (ABI Prism 7300 Detection System, Applied Biosystems). Primer sequences are detailed in Table S1.

### Antibodies and immunoblotting

Western blot analysis was performed on nuclear extracts (Qproteome Cell Compartment Kit, Qiagen). Nuclear fractions were concentrated then quantified using the Bradford assay. Thirty ug of protein was added to each lane and westerns performed with all samples on two separate occasions using HIF-1alpha (1∶1000 dilution) and beta-catenin (1∶2000 dilution) (Novus Biologicals) for 16 h at 4°C. Nuclear loading controls were examined in separate experiments using an antibody directed to the histone associated protein RBAP46 (Cell Signaling) at a dilution of 1∶1000. Secondary antibodies for anti- HIF-1alpha and beta-catenin were from BioRad (1∶2000 dilution) and Invitrogen for anti- RBAP46 (1∶10000 dilution), incubated for 1 hr at room temperature.

### Statistical analysis

Unpaired t-tests were used to compare individual means. Unless otherwise stated data are presented as mean ± standard deviation (SD). Statistical significance defined by P<0.05.

## Supporting Information

Table S1 **Primer Sequences.**
(DOCX)
