# Supplementary material for: Effect of Oxygen on Cardiac Differentiation in Mouse iPS Cells: Role of Hypoxia Inducible Factor-1 and Wnt/Beta-Catenin Signaling
Source: PLoS One. 2013 Nov 12;8(11):e80280. doi: 10.1371/journal.pone.0080280 (PMC3827186; doi:10.1371/journal.pone.0080280)
Supplement: Table S1 — Primer Sequences. (DOCX) [file pone.0080280.s001.docx]

### Table S1. Primer sequences.

|  | **Gene** | | **Forward Primer** | | **Reverse Primer** |
| --- | --- | --- | --- | --- | --- |
| **RT-PCR** | | | | | |
|  | | Sox2 | TAGAGCTAGACTCCGGGCGATGA | TTGCCTTAAACAAGACCACGAAA | |
|  | | Oct3/4 | AGCACGAGTGGAAAGCACT | CTCATTGTTGTCGGCTTCCT | |
|  | | Nanog | AGGGTCTCGTACTGAGATGCTCTG | CAACCACTGGTTTTTCTGCCACCG | |
|  | | GAPDH | AGAACATCATCCCTGCATCC | CCTGCTTCACCACCTTCTTG | |
| **Real-time RT-PCR** | | | | | |
| Transgenes | | Oct3/4 | GCTCTCCCATGCATTCAAAC | | CCCTTTTTCTGGAGACTAAATAAA |
|  | | Sox2 | ATGGCCCAGCACTACCAGAG | | CCCTTTTTCTGGAGACTAAATAAA |
|  | | c-Myc | TTGAGGAAACGACGAGAACAG | | CCCTTTTTCTGGAGACTAAATAAA |
|  | | Klf4 | GGACCACCTTGCCTTACACA | | CCCTTTTTCTGGAGACTAAATAAA |
| Endogenous | | Oct3/4 | GCTCTCCCATGCATTCAAAC | | TGTCTACCTCCCTTGCCTTG |
|  | | Sox2 | ATGGCCCAGCACTACCAGAG | | CTTCTCCAGTTCGCAGTCCA |
|  | | c-Myc | AGCACAAGCTCACCTCTGAAA | | GCTCCTCCTCGAGTTAGGTCA |
|  | | Klf4 | CAGTGCCAGAAGTGTGACAGG | | TCGTGGGAAGACAGTGTGAA |
|  | | Nanog | TTTGGAAGCCACTAGGGAAAG | | AAGCCCAGATGTTGCGTAAGT |
| Cardiac | | Brachyury | ATAACGCCAGCCCACCTACT | | TGGTACCATTGCTCACAGACC |
|  | | Isl-1 | CGTGAAATTCTGGGTCTCTTAAGC | | GGAGGAGAGGCAAACGTAAAAG |
|  | | Tnnc1 | CAGCAAAGGGAAGTCTGAGG | | TGCAGCATCATCTTCAGCTC |
|  | | βMHC | GCATTCTCCTGCTGTTTCCTT | | TGGATTCTCAAACGTGCTTAGTG |
|  | | HIF-1α | CATGATGGCTCCCTTTTTGA | | GTCACCTGGTTGCTGCAATA |
|  | | Mef2C | GCGCTCCACCTCGGCTCTGT | | GGGTGGTGGTACGGTCTCCCA |
|  | | Tbx5 | GGAAAGATGAGGAATGTTCCAG | | GTGTTACAGCTGATGTCCTCCA |
|  | | Titin | TTGAAATCCGAAATGCGGCTG | | TCTTCCACCAGAGGGAGCAC |
|  | | Cav1.2 (Cacna1c) | CATGAAGCTCAACTCAACTGTTTC | | CGTGGGCTCCCATAGTTG |
|  | | Kcnh2 | GATCGCCTTCTACCGGAAA | | CATTCTTCACGGGTACCACA |
